# Supplementary material for: Human visual grouping based on within- and cross-area temporal correlations
Source: PLoS Comput Biol. 2025 Sep 11;21(9):e1013001. doi: 10.1371/journal.pcbi.1013001 (PMC12440224; doi:10.1371/journal.pcbi.1013001)
Supplement: S1 Appendix — (DOCX) [file pcbi.1013001.s001.docx]

Supplementary data


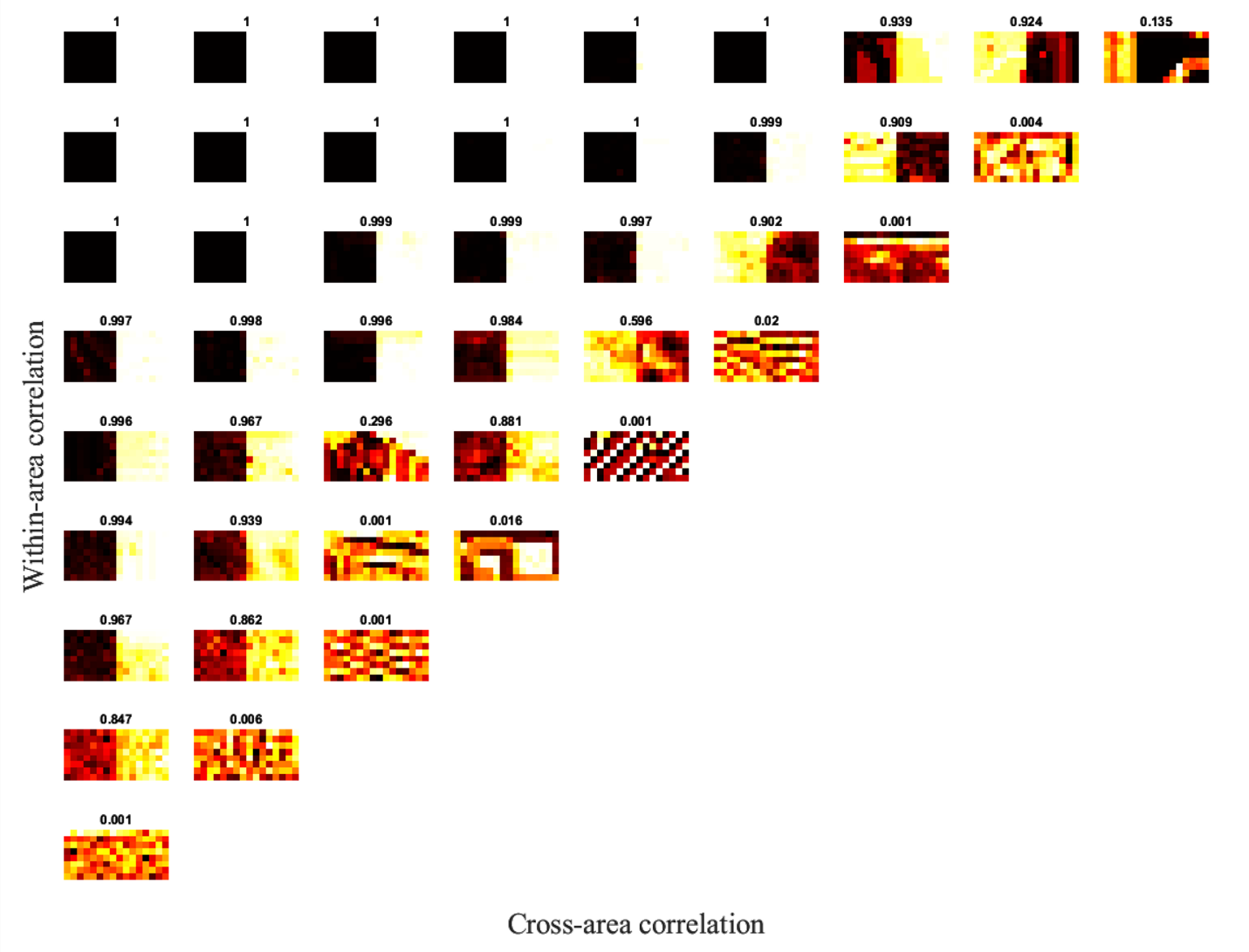
Appendix1. Demo output with the signal interval input from the eigendecomposition of the graph Laplacian in the Naïve model. Each of the graphs represents one demo of the output without any supervision of the answer; each column stands for cross-area correlation from left to right for 0.1 to 0.9, while each row stands for within-area correlation from bottom to top for 0.1 to 0.9. The number above each graph is the correctness rate we defined in the article; the higher the value, the closer to the ground truth.
