# Supplementary material for: Human visual grouping based on within- and cross-area temporal correlations
Source: PLoS Comput Biol. 2025 Sep 11;21(9):e1013001. doi: 10.1371/journal.pcbi.1013001 (PMC12440224; doi:10.1371/journal.pcbi.1013001)

Appendix2. Demo output with the null interval input from the eigendecomposition of the graph Laplacian in the Naïve model.
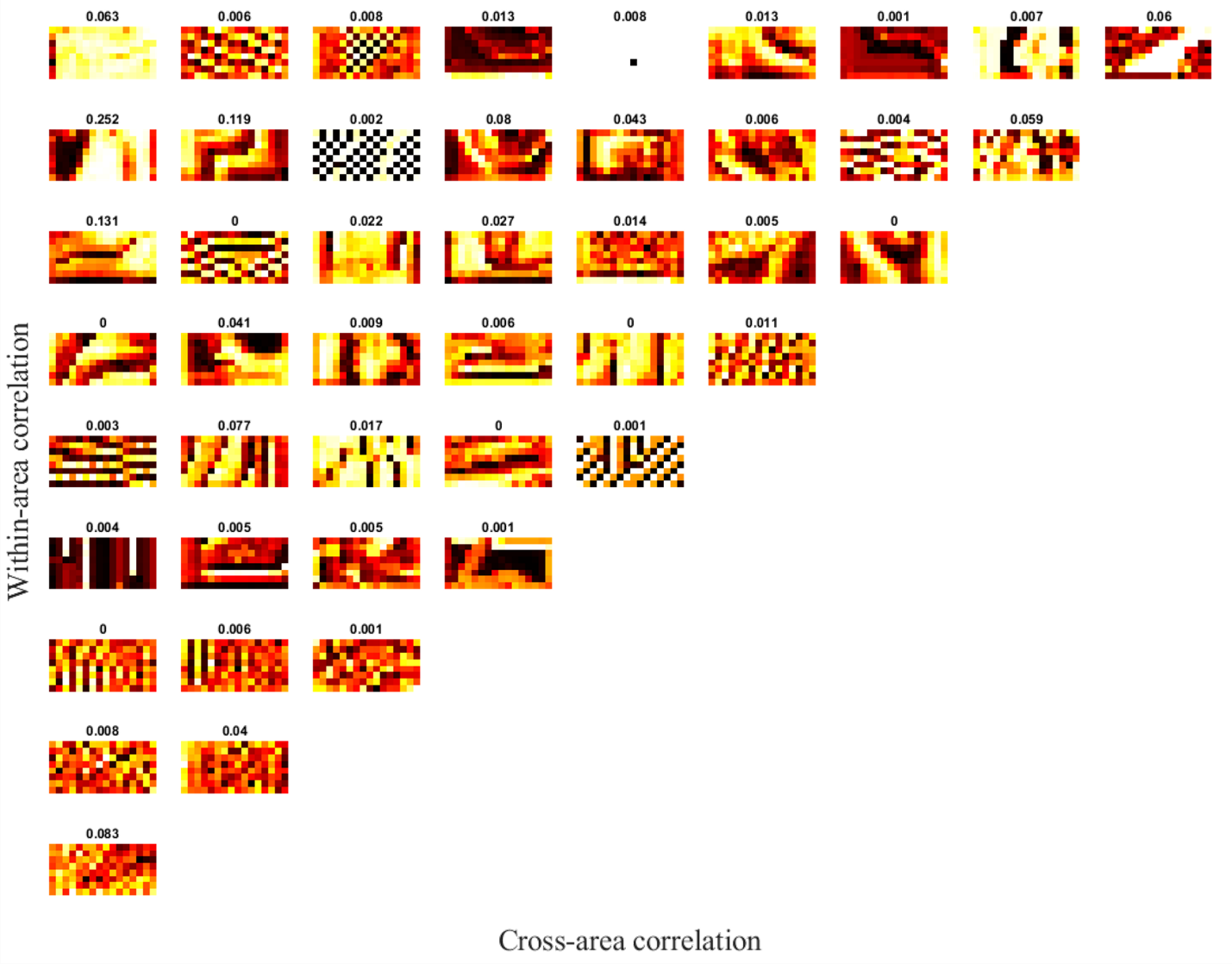

Supplement: S2 Appendix — (DOCX) [file pcbi.1013001.s002.docx]
