# Supplementary material for: Human visual grouping based on within- and cross-area temporal correlations
Source: PLoS Comput Biol. 2025 Sep 11;21(9):e1013001. doi: 10.1371/journal.pcbi.1013001 (PMC12440224; doi:10.1371/journal.pcbi.1013001)
Supplement: Attachment — Submitted filename: Point to Point Response_ver3-1.docx [file pcbi.1013001.s004.docx]

# To Reviewer 1

First, and most importantly, I think the paper would be improved by making the questions addressed in Studies 2 and 3 of the study earlier and more clearly. In particular, after Study 1 (p. 15), the authors go very quickly into experimental parameters for Study 2. This reader would have been helped by a more thorough explanation of what was being tested and what the relevant hypotheses were going into Study 2.

Response:

We thank the reviewer for the helpful suggestion. We have added a clear explanation of the experimental purpose, motivation, and hypotheses at the beginning of Study 2.

For Study 3, I was a little unclear about whether the naïve model was the best foil to compare with the parameterized model. It seems predictable that a model that directly computes cosine similarity will perform near-optimally and make few humanlike errors. Under what hypothesis would human performance match the naïve model better or equally well to the parameterized model? Is there another model, potentially a non-graph cut model whose differences in human predictability would be more illuminating?

Response:

The goal of Study 3 was to test whether a global segmentation algorithm based purely on temporal similarity structure (both within- and cross-area correlations) could account for human performance. Our intention was not to claim that the human visual system uses the specific algorithm introduced here. We believe that other algorithms that utilize a global similarity structure for optimal region segregation, as does the graph-cut algorithm, could also be candidates for models of human segregation computation. Our focus is more on the computational constraints than on the algorithm the visual system uses to solve them.

Within this context, the naïve model serves as a model close to an ideal observer, showing the upper-bound performance of the graph-cut algorithm when no biological limitations are imposed. We agree that human observers are unlikely to reach the performance of this model. The main point of our argument is that by simply adding reasonable biological factors (noise and spatial pooling tuning) to the naïve model, the graph-cut model can explain the human data reasonably well. We added a brief description to explain that the naïve model is just for showing the upper limit of this graph-based algorithm.

It should also be noted that most of the existing models for texture segregation in vision science are designed for static image segmentation, and there are currently no well-established models for temporal similarity–based segmentation. This makes it difficult for us to identify a meaningful non-graph-cut model for comparison.

Finally, I think the authors could make clearer what was learned about human perception in the General Discussion. The GD does have some of this, and I like what is written, but given the breadth of readership at PLOS CB, I think many readers would benefit from a little additional explanation of the key takeaways

Response:

We thank the reviewer for the helpful suggestion. We have added a brief concluding paragraph at the end of the General Discussion to highlight the key contributions and perceptual insights of the study.

On page 6 (first bullet point), the authors say that W is short for “weight”. Corrrect me if I’m wrong, but should this be “width”?

Response:

We appreciate the careful reading. The reviewer is correct — “W” refers to “width”, not “weight”. We have corrected the text accordingly in the revised manuscript.

# To Reviewer 2

The stimuli generation methods (Exp1). I found a lot of details are missing. First, I think I understand that each frame is in-distinguishable between areas, the temporal information allows such distinguish, am I right? However, I am a bit worried by the consistant and clear cut off between the two areas (easy to calculate).

For instance, a recent paper (Vacher, J., Launay, C., Mamassian, P., & Coen-Cagli, R. (2023). Measuring uncertainty in human visual segmentation. PLoS Computational Biology, 19(9), e1011483.) tested a similar research question, but do not have such a rigorous and pre-defined boundary (or box).

Response:

Yes, your understanding is correct — we aimed to remove all static visual cues so that the stimuli would be indistinguishable in individual frames and only separable based on temporal information.

We appreciate your comment and the reference to Vacher et al. (2023). In our study, we used a fixed and simple segmentation boundary because our focus was on how different within- and cross-region correlations affect spatial grouping performance. This setup allowed us to isolate and precisely control the temporal similarity structure. We acknowledge, however, that this design does not allow us to assess the segmentation under more naturalistic situations. We have added a note in the General Discussion to clarify this limitation and suggest that future work could combine our stimulus generation method and Vacher’s perception measurement method to better characterize the mechanism of segmentation based on temporal correlations.

The human data collection needs more details. (1) How many subjects? The authors said there are 4 (2 authors + 2 naive subjects), however, there are 5 participants' data in Figure 5 (excluding the 'general' one). I think this is very concerning. (2) Details of the procedure. This Expiement 2 is very long (7.5 hrs), pls specify the duration of each block and the gap/rest/interval between for future replication. (3) The major concern lies in here: if half of the subjects are the authors, even if they cant figure out the 'correct answer' simply from images (nice image generation from Exp 1), they are still aware the stimuli were seperated at the centre. SO it is very possible for them (which consistute half of the data!) to simply rely on the centre part of the videos/stimuli (not necessarily the whole image) to make the judgement. Yes, the stimuli are so 'uniform' in this away.

Moreover, the design of Exp 2 is a bit off, why 2IFC? Is it possible that both videos are very much seperable but they have to chose one, or vice versa? Some additional details are necessary.

Response:

(1) We thank the reviewer for pointing this out. There was a typo in the original text. As shown in Figure 5, the dataset includes two author participants (ZTS and YJC) and three naïve participants (TI, YL, ZWH). We have corrected the manuscript accordingly.

(2) We have reorganized the description of the human experiment procedure to provide a concise but complete account, including block structure and timing details to facilitate reproducibility for future studies.

(3) As shown in Figure 5, the overall trends in the performance data from the author participants are comparable to those of the naïve participants. There is no apparent deviation that would suggest prior knowledge of the detailed stimulus generation process affected response patterns. Note, however, that the naïve participants were instructed about the stimulus configuration before the experiment started.

Since we fixed the region boundary, it is probable that our participants, including naïve ones, paid attention to the centre part of the stimulus. However, our data indicate that not only short-range cross-area correlations across the boundary, but also long-range within-area correlations affect the participants segregation judgments. This is indicated by the model simulation result shown in Fig. 8 that the parameter determing the range of spatial interactions (sigma) should not be small to account for the human data. The successful segmentation requires integrating temporal similarity across multiple element pairs, including those at relatively distant locations.

We acknowledge that determining whether boundary-local correlations receive greater weight when the boundary location is known, and how the visual system initially infers boundary locations, are questions beyond the scope of this study. These points are discussed in the General Discussion as directions for future research using more complex, non-uniform spatial configurations.

In response to the comment about the 2IFC task design, we have clarified in the revised manuscript that the signal and null intervals are systematically different: the signal interval is designed to be segmentable based on temporal correlation structure, while the null interval is not. When both intervals appear similarly unsegmentable, they should look very similar, since they were matched in the within-region correlation.

The Exp3's data suggests the SNR is an important factor, see minor concern for more details. I am a bit worried (as the authors stated in discussion), even this model could not read the 'temporal' aspect and so the stimuli are flatted from 3D to 2D. Does this affect the interpretation of the data?

Response:

We believe the reviewer may be referring to the transformation described in Equation (17). This flattening from 3D to 2D was done purely for computational convenience and does not result in any loss of spatial or temporal information. The similarity matrix is computed based on the temporal sequence at each spatial location, meaning the model explicitly processes temporal signals when constructing the similarity structure.

Behavioural data suggests the seperation ratios are “Luminance > Colour > Phase”.

The difference is compatible with known channel SNR differences (as mentioned in Exp3), yet the manuscript currently leaves the reader wondering if uncontrolled cues leaked through stimulus generation. A discussion or justification is necesary.

Response:

We appreciate the reviewer’s thoughtful concern. First, we clarify that the same set of 30 pre-generated temporal tensors was used across all three attributes (luminance, color, and spatial phase), ensuring there were no physical differences in the underlying temporal structure across conditions. Second, as detailed in Study 1, we confirmed that the spatial mean and contrast between the two regions were either matched or negligible, making static segmentation cues unavailable for all attributes. Third, the temporal spectrum of the stimuli was approximately white noise, which ensures broad activation of the relevant visual channels regardless of their specific tuning characteristics.

Given these controls, the observed performance differences are most likely due to intrinsic differences in signal strength across attributes. As noted in the discussion, the spatial phase condition likely suffers from lower overall signal strength due to its narrowband and low-sensitivity temporal tuning. We have added a brief clarification in the General Discussion to reflect this interpretation.

Does one need the ViT to create the stimuli? Is it possible we can create these stimuli with a rigorous pre-defined algorithm? Does the ViT neccesary here?

Response:

We do not claim that the ViT-based generator is the only possible solution. Rather, it is one practical approach that meets the specific needs of this study—namely, precise and efficient control of dense pair-wise temporal correlations across many elements. Other carefully designed algorithms might achieve similar goals, and we welcome future work exploring such alternatives. In our case, the ViT framework provided a straightforward way to optimize correlations while maintaining generation efficiency and numerical stability, which is why we adopted it here.

# To Reviewer 3

In many places the authors talk about similarity or correlation, they must precise that it is temporal (or not) ! (eg l461)

Response:

All instances of "correlation" in the manuscript refer to temporal correlation. We have reviewed the text and confirmed that terminology is used consistently throughout. We appreciate the reviewer’s attention to this detail.

Section Design and procedure (l383) : the writing must be improved/simplified. I had the impression to read the same info twice written in slightly different manner.

The section contain redoundancies :

- "introduce three different attributes" / "we examine three attributes"

- "viewed two consecutive 1-second video clips" / "view 1.000 ms video clips"

Response:

Following this suggestion, we have reorganized and revised the description of the human experiment design and procedure to provide a more concise yet complete explanation.

The number of repetitions for each of the 45 cor pairs is not indicated.

Response:

This comment is partially addressed in the previous response. We have reorganized the experiment description and added the number of repetitions for each correlation pair. The number of simulation repetitions (i.e., 1000) has also been specified in the model section.

Typos :

l51 : computational model

l307 : missing ref for figure

l374 : it seems you have 5 participants not 4

l513 : it's unclear what is the normalized Laplacian matrix

l527 : \bar T is the target not T(h,w)

Response:

We thank the reviewer for the detailed checks. All typographical errors have been corrected. A brief description of the normalized Laplacian matrix has been added following its formal definition to improve clarity.

Regarding the expression$\bar{\mathbb{T}}$, we would like to clarify that it does not refer to the entire target matrix. Instead, $\bar{\mathbb{T}}$ is not the entire target itself. We use  $\bar{\mathbb{T}}$ as part of a mean-centering procedure before computing the performance index. We have added a sentence to the manuscript to clarify this point.

- Eq 9 why absolute values instead of squared ? Eq 11 why norm-1 is used ? Isn't it a regular euclidean norm ? (in fact he question hold for all norms !)

Response:

The use of the L1 norm was arbitrary, and in theory, the results should not differ significantly from those using the L2 norm. We chose the L1 norm for consistency across loss components and because it tends to be more robust to noise. Additionally, as shown in Study 1, we conducted explicit validation of the generated stimuli to ensure that the quality was sufficient for the intended manipulations.

- Figure 5 : why the checkerboards are not straight ? It is unclear what these weird shapes are representing.

Response:

We thank the reviewer for the comment. The axes in Figure 5 are based on the empirical cosine similarity values of the generated stimuli, rather than idealized theoretical values (e.g., 0.1, 0.2, …, 0.9). Due to slight deviations in the actual similarity values, the grid appears uneven. This is expected and has been noted in the figure caption.

Complementary references for discussion

- Vacher, J., Launay, C., Mamassian, P., & Coen-Cagli, R. (2023). Measuring uncertainty in human visual segmentation. PLoS Computational Biology, 19(9), e1011483

- Wallis, Thomas SA, et al. "Image content is more important than Bouma’s Law for scene metamers." ELife 8 (2019): e42512.

Response:

We thank the reviewer for the suggestion. The cited references have been added to the revised manuscript.
