# Supplementary material for: Human visual grouping based on within- and cross-area temporal correlations
Source: PLoS Comput Biol. 2025 Sep 11;21(9):e1013001. doi: 10.1371/journal.pcbi.1013001 (PMC12440224; doi:10.1371/journal.pcbi.1013001)
Supplement: Attachment — Submitted filename: Point to Point Response_Rev2_Ver1.docx [file pcbi.1013001.s005.docx]

# To Reviewer 1

p. 7: "In our implementation, we used several

188 four stacked ResidualBlock2D modules, each consisting of convolution, normalization

189 (InstanceNorm2D), and GELU activation, with the residual connection between two layers." Should be corrected

Response:

Thank you for pointing this out. We have corrected the sentence to avoid ambiguity. The revised sentence now reads:

“In our implementation, we used four-stacked ResidualBlock2D modules, each consisting of convolution, normalization (InstanceNorm2D), and GELU activation, with the residual connection between two layers.”

p. 17: As reviewer 2 pointed out, the demographic information is not complete. There were 5 participants, but only four participants' demographic information was reported.

Response:

We apologize for the oversight. We have corrected the demographic information to include all five participants in the revised manuscript.

# To Reviewer 2

But I still want to point out that in the current human visual cognition/visual perception studies, we tend to have a larger sample size. While I believe the authors carried over some tendency from animal (primates) studies (like testing a lot of trials on two monkeys), I still suggest that they carefully note this limitation in the discussion.

Response:

We thank the reviewer for the suggestion. We have added a note in the General Discussion to acknowledge that the small participant number may limit the generalizability of temporal segmentation based on temporal correlation, and we mentioned that larger participant groups are required for future studies.

# To Reviewer 3

I thank the authors for their responses and I have no remaining remarks. I think the paper is ready for publication.

Response:

We sincerely thank the reviewer for the positive evaluation and supportive comments.
